# DDX3X Links NLRP11 to the Regulation of Type I Interferon Responses and NLRP3 Inflammasome Activation

**DOI:** 10.3389/fimmu.2021.653883

**Published:** 2021-05-13

**Authors:** Ioannis Kienes, Sarah Bauer, Clarissa Gottschild, Nora Mirza, Jens Pfannstiel, Martina Schröder, Thomas A. Kufer

**Affiliations:** ^1^ Department of Immunology, Institute of Nutritional Medicine, University of Hohenheim, Stuttgart, Germany; ^2^ Core Facility University of Hohenheim, Mass Spectrometry Module, University of Hohenheim, Stuttgart, Germany; ^3^ Kathleen Lonsdale Institute for Human Health Research, Department of Biology, Maynooth University, Maynooth, Ireland

**Keywords:** innate immunity, nod-like receptors, anti-viral, DEAD-box helicase, inflammasome, IL-1, type I interferon

## Abstract

Tight regulation of inflammatory cytokine and interferon (IFN) production in innate immunity is pivotal for optimal control of pathogens and avoidance of immunopathology. The human Nod-like receptor (NLR) NLRP11 has been shown to regulate type I IFN and pro-inflammatory cytokine responses. Here, we identified the ATP-dependent RNA helicase DDX3X as a novel binding partner of NLRP11, using co-immunoprecipitation and LC-MS/MS. DDX3X is known to enhance type I IFN responses and NLRP3 inflammasome activation. We demonstrate that NLRP11 can abolish IKKϵ-mediated phosphorylation of DDX3X, resulting in lower type I IFN induction upon viral infection. These effects were dependent on the LRR domain of NLRP11 that we mapped as the interaction domain for DDX3X. In addition, NLRP11 also suppressed NLRP3-mediated caspase-1 activation in an LRR domain-dependent manner, suggesting that NLRP11 might sequester DDX3X and prevent it from promoting NLRP3-induced inflammasome activation. Taken together, our data revealed DDX3X as a central target of NLRP11, which can mediate the effects of NLRP11 on type I IFN induction as well as NLRP3 inflammasome activation. This expands our knowledge of the molecular mechanisms underlying NLRP11 function in innate immunity and suggests that both NLRP11 and DDX3X might be promising targets for modulation of innate immune responses.

## Introduction

In mammals, viral infections are detected by anti-viral pattern-recognition receptors (PRRs), including RIG-I-like receptors (RLRs), cytosolic DNA receptors, and endosomal Toll-like receptors (TLRs). Their activation induces antiviral cytokine responses dominated by release of type I interferons (IFNs), which are crucial for limiting replication of most viruses (1–4). Hence, failure to initiate an effective IFN response correlates with higher pathogenicity in many viral infections (5–7). Thus, robust early IFN induction is crucial for controlling viral replication, but failure to resolve this response can result in severe immunopathology in the host (8–10). In particular an altered balance between type I IFN responses and pro-inflammatory cytokine release can lead to pathology in the host, as seen for example in COVID-19 disease (11, 12). It is therefore critical to understand how type I IFN and pro-inflammatory cytokine responses are balanced during viral infections.

RLRs, most prominently RIG-I, are essential sensors for cytosolic viral RNA (13). Following the binding of viral RNAs, RIG-I oligomerizes and binds to the mitochondrial antiviral signaling protein (MAVS) *via* CARD-CARD interactions (14). MAVS then recruits downstream signaling proteins, such as TNF-associated factor 2 (TRAF2) (15), TRAF6 (16), and TRAF3 (17), which ultimately lead to induction of pro-inflammatory cytokines and type I IFNs. IFN induction is dependent on phosphorylation of the transcription factors interferon regulatory factor 3 (IRF3) and IRF7 (18–21) mediated by two related kinases, inhibitor of NF-κB kinase subunit epsilon (IKKϵ) and TANK binding kinase 1 (TBK1) (22, 23). TBK1 is necessary for IFN-β induction and ubiquitously expressed in different cell types, while IKKϵ may not be essential for IFN-β induction and has been suggested to directly regulate a subset of interferon-stimulated genes (ISGs) (24).

NOD-like receptors (NLRs) are another important group of PRRs and a total of 22 human NLR proteins have been discovered (25), many of which remain to be functionally characterized. NLRs consist of an N-terminal effector domain, a central NACHT domain, and a C-terminal leucine-rich repeat region (LRRs), and can be subcategorized by their effector domains into pyrin-domain (PYD) containing NLRP proteins, CARD-domain containing NLRC proteins, baculovirus inhibitor of apoptosis (BIR)-domain containing NLRB proteins, and CARD-transcriptional activation-domain (CARD-AD) containing NLRA proteins (26).

Signaling pathways induced by NLRs are heterogeneous. While NOD1 and NOD2 induce NF-κB activation upon recognition of bacterial ligands (27–29), NLRP1, NLRP3 and NLRC4 activation leads to formation of a large multiprotein signaling platform called the inflammasome (30–32). Inflammasome formation starts with recruitment of the adaptor protein apoptosis-associated speck-like protein (ASC), which then recruits and activates pro-caspase-1, enabling maturation and release of IL-1β and IL-18. Other NLRP proteins including NLRP6, NLRP7, NLRP12, and possibly NLRC5 might also form inflammasomes (33–36). However, not all mammalian NLR proteins act as PRRs. For example, NLRC5 and CIITA are transcriptional enhancers for MHC class I and class II genes, respectively (37, 38), and some NLRs have been identified as negative regulators of innate immune responses. An example is NLRC3 that negatively regulates DNA sensing-PRRs by interfering with the adaptor molecule stimulator of interferon genes (STING) (39). Furthermore, NLRP4 (40–42), NLRP12 (43, 44) and NLRP14 (45), were reported to modulate IFN responses (for an overview see (46)).

We and others have previously shown that NLRP11 can negatively regulate both NF-κB activation (47) and type I IFN induction (48, 49). It was shown that NLRP11 interferes with the MAVS signaling complex (47, 49), but it can also block type I IFN induction downstream of TBK1 (48), suggesting that NLRP11 might intervene at multiple levels in the RLR pathway.

A well-established positive regulator of the RLR-pathway is the human DEAD-box protein 3 (DDX3X). DDX3X physically interacts with MAVS (50), IKKϵ (51), TBK1 (52), TRAF3 (53) and IRF3 (54), resulting in enhanced type I IFN production (50, 51, 53, 54). DDX3X might also be directly involved in recognition of viral RNA (50). Activation of the RIG-I pathway triggers binding of DDX3X to IKKϵ, which leads to enhanced IKKϵ activation (51, 54) and IKKε-mediated phosphorylation of DDX3X in its N-terminal region, which enables recruitment of IRF3 into the complex, resulting in enhanced activation of IRF3 by IKKϵ phosphorylation (54). TBK1 can also phosphorylate DDX3X and enhance IFNβ production (52). The physiological importance of DDX3X’s role in anti-viral immune signaling is underlined by the fact that several viruses, including Vaccinia virus, Hepatitis B virus and Influenza A virus, evolved immune evasion mechanisms targeting DDX3X (51, 55–59). Thus DDX3X is a central regulator of the RIG-I anti-viral signaling pathway, where it interacts with signaling intermediates in a complex manner that is still not completely understood.

Recently, a further role for DDX3X as a positive regulator of NLRP3 inflammasome activation was reported. Sequestration of DDX3X into cytosolic stress granules during cellular stress results in reduced NLRP3/caspase-1 activation and suppression of IL-1β and IL-18 release (60).

A better understanding of the complex molecular circuits that control innate immune responses will advance our understanding of host-pathogen interactions and help to identify novel targets for therapeutic intervention. Here we provide evidence that NLRP11, *via* its leucine-rich repeats (LRRs), interacts with DDX3X. This interaction inhibited IKKϵ-induced phosphorylation of DDX3X and type I IFN induction. Using caspase-1 activation assays and ASC speck formation assays, we revealed that NLRP11 can also counteract the positive effect of DDX3X on NLRP3 inflammasome activation. Taken together, our work identified DDX3X as a novel target of NLRP11 that contributes to the inhibitory effects on both type I IFN induction and IL-1β release.

## Materials and Methods

### Plasmids and Reagents

Myc-NLRP11 and myc-NLRP11 domain constructs were previously described (48). The plasmids pcDNA5/FRT/TO-NLRP11-eGFP and pEGFP-DDX3X were generated by molecular cloning from myc-NLRP11 or myc-DDX3X respectively. The Myc-DDX3X construct is described in (51). piGLuc caspase-1 reporter plasmid, pCI-caspase-1 and pCI-ASC-HA were kindly provided by Veit Hornung and are described in (61, 62). FLAG-IKKϵ was kindly provided by Eliane Meurs (63). Myc-DDX3X mutants were previously described in (54). All plasmids (inserts, tags and flanking regions) were verified by Sanger sequencing.

### Cell Culture

HEK293T cells (ATCC, CRL-3216) were grown in DMEM supplemented with 10% heat-inactivated FBS. HEK Blue IFN-α/β (hkb-ifnab, InvivoGen) were maintained in DMEM, supplemented with 10% heat-inactivated FBS, 30 μg/ml blasticidin and 100 μg/ml zeocin. THP1 cells with a doxycycline inducible shRNA targeting DDX3X and non-silencing controls (NSC) are described in (64). Cells were grown in RPMI 1640 supplemented with 10% heat-inactivated FBS, gentamycin and puromycin. Knock-down of DDX3X was induced by 1 µg/ml doxycycline for 48 h.

Stable, inducible cell lines expressing NLRP11-eGFP were generated by co-transfection of pOG44 and pcDNA5/FRT/TO-NLRP11-eGFP at 9:1 ratio into Flp-In T-REx HEK293 (Invitrogen/ThermoFischer, R78007) and HeLa FlpIn T-REx cells (kindly provided by the Hentze Lab, EMBL Heidelberg) using Lipofectamin 2000 (Thermo Fisher Scientific) and selected with 10 µg/ml blasticidin and either 100 µg/ml (HEK) or 600 µg/ml (HeLa) hygromycin. Single clones were selected, and expression was induced by 1 µg/ml doxycycline for at least 16 h prior to further experiments. All cell culture media were supplemented with penicillin and streptomycin. Cells were routinely monitored for absence of mycoplasma infection by PCR.

For viral infection, cells were incubated with 160 hemagglutination units (HAU)/ml Sendai virus (Cantell Strain in allantoic fluid, Charles River).

### siRNA- and shRNA Mediated Silencing

THP1 and THP1shDDX3X cells were differentiated with 100 nM PMA for 16 h. Medium was changed and cells were incubated for 24 h prior to siRNA-mediated knock-down with 100 nM siRNA, transfected using HiPerFect transfection reagent (Qiagen) according to (65). AllStars negative control siRNA and siNLRP11_6 CACGACCTTGCAGCTGTCGAA (48) (Qiagen) were used. Knock-down of NLRP11 was performed for 72 h. For double knock-down of NLRP11 and DDX3X, 24 h after transfection of the siRNA, THP1 shDDX3X cells were induced with 1 µg/ml doxycycline for 48 h.

Knock-down efficiency of NLRP11 was monitored with endpoint PCR as described in (48). Knock-down of DDX3X was monitored by end-point PCR using the following primer pair: fwd: TGCTGGCCTAGACCTGAACT rev: TTGATCCACTTCCACGATCA.

### Co-Immunoprecipitation and Protein Binding Assays

Co-immunoprecipitation of NLRP11-eGFP, from HEK293 and HeLa FlpIn eGFP and NLRP11-eGFP cell lines, or of eGFP-DDX3X from HEK293T cells, transiently transfected with Lipofectamine 2000 (Thermo Fisher Scientific), was performed with GFP-Trap Agarose resin (Chromotek). Cells were lysed in Triton buffer [50 mM Tris/HCl pH 7.4, 150 mM NaCl, 1% Triton-X100, 1% Na-Deoxycholate, 100 nM β-glycerophosphate, 100 nM sodium orthovanadate, 1 mM NaF and cOmplete Mini Protease inhibitor Cocktail (Roche)]. Lysates were cleared by centrifugation (15 min, 4°C, 21000 x *g*) before the supernatants were loaded onto the matrix. Precipitation was performed at 4°C for 3 h, before the matrix was washed with washing buffer (50 mM Tris/HCl pH 7.4, 150 mM NaCl, 1% Na-Deoxycholate).

### NanoLC-MS/MS Analysis

Proteins were digested on beads using trypsin (Roche, Germany) in 6 M urea, 50 mM Tris-HCl pH 8.5. Cysteines were reduced using 1,4-dithiothreitol (DTT) and then alkylated by chloroacetamide. Samples were then diluted to a final concentration of 2 M urea. 750 ng trypsin were added, and samples were digested overnight at 25°C. The digests were stopped by adding trifluoroacetic acid (TFA). Next, peptide mixtures were concentrated and desalted on C18 stage tips and dried under vacuum. Samples were dissolved in 0.1% TFA and were subjected to nanoLC-MS/MS analysis on an EASY-nLC 1000 system (Thermo Fisher Scientific, Germany) coupled to a Q-Exactive Plus mass spectrometer (Thermo Fisher Scientific, Germany) using an EASY-Spray nanoelectrospray ion source (Thermo Fisher Scientific, Germany). The system was controlled by Xcalibur 3.0.63 software. Tryptic peptides were directly injected to a 25 cm x 75 µm EASY-Spray analytical column (2 μm, 100 Å, PepMap C18) operated at 35°C. Peptides were separated at a flow rate of 250 nL/min using a 120 min gradient with the following profile: 3% - 10% solvent B in 50 min, 10% - 22% solvent B in 40 min, 22% - 45% solvent B in 30 min, 45% - 90% solvent B in 10 min, 15 min isocratic at 90% solvent B, followed by 90% - 3% solvent B in 10 min and re-equilibration at 3% solvent B for 15 min. (solvent A: 0.5% acetic acid; solvent B: acetonitrile/H_2_O (80/20, v/v), 0.5% acetic acid).

MS spectra (m/z = 300-1600) were detected at a resolution of 70000 (m/z = 200) using a maximum injection time (MIT) of 100 ms and an automatic gain control (AGC) value of 1 × 10^6^. MS/MS spectra were generated for the 10 most abundant peptide precursors using high energy collision dissociation (HCD) fragmentation at a resolution of 17500, normalized collision energy of 27 and an intensity threshold of 1.3 × 10^5^. Only ions with charge states from +2 to +5 were selected for fragmentation using an isolation width of 1.6 Da. For each MS/MS scan, the AGC was set at 5 × 10^5^ and the MIT was 100 ms. Mascot 2.6 (Matrix Science, UK) was used as search engine for protein identification. Spectra were searched against the human UniProt database (66). Scaffold 4.8.6. (Proteome Software, USA) was used to evaluate peptide identifications. These were accepted with a peptide probability greater than 80% as specified by the Peptide Prophet algorithm (67). Proteins had to be identified by at least one unique peptide and a protein probability of at least 99% to be accepted.

### Reporter Assays

For iGLuc caspase-1 reporter assays, HEK293T cells were seeded in a 96-well plate (Greiner) at a density of 35,000 cells per well and transiently transfected using Xtreme Gene 9 transfection reagent (Sigma Aldrich) with 8.6 ng β-galactosidase plasmid, 42 ng of the iGLuc reporter plasmid (61), and expression plasmids of NLRP3, NLRP11, ASC, caspase-1 and DDX3X, DDX3X S102A, or DDX3X 4A as indicated. 20 h after transfection, cells were stimulated with 15 μM nigericin (InvivoGen) for 3 h. Cells were then lysed in 100 µl passive lysis buffer (Promega) per well. 50 µl of the cell lysate were transferred into a non-transparent 96-well plate. Luciferase activity was measured in a multiplate reader (Enspire, PerkinElmer LifeSciences) after addition of 100 μl of 3.33 μM Coelenterazine (Carl Roth) per well. 100 µl of 1 mg/ml O-nitrohpenyl-β-D-galactoryranoside in 60 mM Na_2_HPO_4_, 40 mM NaH_2_PO_4_, 10 mM KCl and 1 mM MgSO_4_ were added to the remaining 50 µl lysate, incubated at 37°C and absorption was measured at 405 nm (620 nm reference) as β-galactosidase activity. Luciferase activity was normalized to β-galactosidase activity.

*Ifnβ* luciferase reporter assays were performed as described in (48), with 5 ng FLAG-IKKϵ plasmid for activation.

Assays were performed in technical triplicates and repeated independently three times unless indicated otherwise.

### Indirect Immunofluorescence

HeLa FlpIn cells were seeded into 24-well plates at a density of 75,000 cells per well on glass coverslips, HEK FlpIn cells at 100,000 cells per well on poly-L-lysin pretreated glass coverslips and expression of eGFP or eGFP-NLRP11 was induced with 1 µg/ml doxycycline. After overnight expression, cells were either infected with 160 hemagglutination units (HAU)/ml Sendai virus (SeV) for different durations, or directly fixed with 4% PFA in PBS, permeabilized with 0.5% Triton X-100 and blocked with 5% FBS in PBS. Cells were then incubated with primary and secondary antibody sequentially. Antibodies used: Primary: DDX3X (A300-474A Bethyl Laboratories), AIF (Cell Signalling # 4642). Secondary: Alexa-546 goat anti-rabbit IgG, Alexa-405 goat anti-mouse (Molecular Probes). DNA was stained with Hoechst 33258 (Sigma). Images were captured with a Leica DMi8 microscope using a HCX PL FL L 40X/0.60 or a HC PL APO 63X/1.40-0.60 OIL objective and processed using the Leica LasX software and ImageJ. For 3D deconvolution, Z-stacks of 4.05 µm depth were captured, with individual planes every 0.2 µm. Blind 3D-deconvolution was performed using the Leica LasX software, performing 10 iterations at a refractive index of 1.52. For quantitative analysis, sample pictures were blinded and counted by eye.

### Immunoblotting

Immunoblotting was performed as described in (65). Antibodies used: β-actin (C-4; Santa Cruz sc-47778), GFP (Roche 11 814 460 001), myc (9E10; Sigma Aldrich M4439), DDX3X (Bethyl Laboratories A300-474A), FLAG (Sigma Aldrich F7425), GAPDH (Santa Cruz sc-25778), pIRF3 (Cell Signalling #29047T).

### Measurement of Cytokines

IL-1β and IFNβ release was measured in cell supernatants by ELISA (DY201, DY814, R&D Systems) according to the manufacturer’s instructions. A bioassay for type I interferons was performed using HEK Blue IFN-α/β cells (Invivogen). HEK Blue IFN-α/β cells were stimulated for 20 h with supernatant from SeV infected THP1 cells and SEAP activity in the supernatant was measured by Quantiblue solution (rep-qbs, InvivoGen) according to the manufacturer’s conditions.

### Statistical Analysis

Data were analyzed by unequal variances t-test (Welch test) and plotted using GraphPad Prism version 7.05. p < 0.05 was regarded as significant.

## Results

### NLRP11 Interacts With the ATP-Dependent RNA Helicase DDX3X

**Figure 1:**
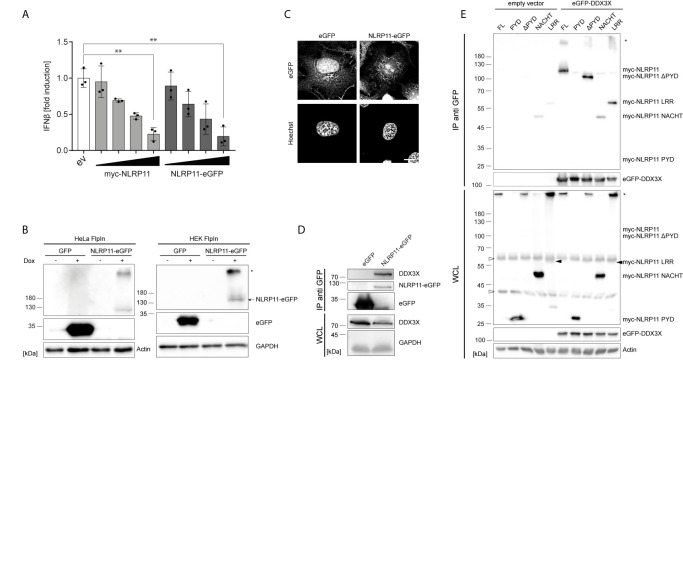
NLRP11 interacts with DDX3X. **(A)** *ifnb* luciferase reporter assay in HEK293T cells overexpressing FLAG-IKKϵ (5 ng) and either empty vector (ev), myc-NLRP11, or NLRP11-eGFP. Means of three independent experiments conducted in triplicates, relative to the mean of 0 ng NLRP11, ± SEM are shown. **p < 0.01; Welch’s two-sided t-test. **(B)** Immunoblot of protein lysates of HEK293- and HeLa-eGFP and -NLRP11-eGFP cells, induced overnight with doxycycline. Probing for GFP and actin, or GAPDH as loading control is shown. Monomeric NLRP11-eGFP is marked by an arrow and aggregated NLRP11 by an asterisk. **(C)** Immunofluorescence micrographs of HeLa-NLRP11-eGFP cells. Cells were induced with doxycycline overnight, fixed and nuclei were stained. 3D-deconvolution of z-stacks of the signal of DNA (lower images) and eGFP (upper images) are shown. Stack size = 0.2 µm. Scale bar = 10 µm. **(D)** Immunoblots from anti-GFP immunoprecipitations (IP) from HEK293T-eGFP and HEK293-NLRP11-eGFP cells induced over night with doxycycline. IPs were probed for DDX3X and GFP, whole cell lysates (WCL) were probed for DDX3X and GAPDH as loading control. Representative blots for two independent experiments are shown. **(E)** Immunoblots from anti-GFP IPs from HEK293T cells expressing empty vector, or eGFP-DDX3X and the indicated myc-NLRP11 construct. IP lysates were probed for myc and GFP, whole cell lysates were probed for myc, GFP and actin as a loading control. Representative blots of two independent experiments are shown. *, NLRP11 aggregate; ∆, unspecific bands.

We recently demonstrated that NLRP11 is a negative regulator of inflammatory cytokine induction (48). In order to study the underlying molecular mechanism, we generated an eGFP-tagged NLRP11 (NLRP11-eGFP) expression construct and confirmed that NLRP11-eGFP retained its negative regulatory effect on IKKϵ induced *ifnβ-*reporter gene expression in comparison to our previously used myc-NLRP11 construct (**Figure 1A**). Stable cell lines allowing for inducible expression of NLRP11-eGFP were generated using the doxycycline inducible single site recombination system Flp-In T-REx. We obtained stable HeLa and HEK293 lines that showed inducible expression of NLRP11-eGFP after doxycycline treatment, as well as control cell lines expressing eGFP only. We selected for cell lines with tight regulation, uniform expression in all cells, and well detectable expression upon induction (**Figure 1B**). We noticed that NLRP11-eGFP tended to form high molecular weight SDS-stable aggregates in both HEK293 and HeLa cells (**Figure 1B**, upper band), which we also observed for myc-NLRP11 (data not shown). However, we always also detected monomeric NLRP11 in SDS-PAGE, and fluorescent microscopy also confirmed that NLRP11-eGFP was distributed in the cytoplasm without formation of larger aggregates, consistent with earlier reports (48, 49). Nonetheless, we noticed that NLRP11 was not completely evenly distributed in the cytosol, suggesting it might associate with cellular organelles (**Figure 1C**).

Next, we used these stable cell lines to identify interaction partners of NLRP11. NLRP11-eGFP protein complexes were immunoprecipitated from the HeLa-NLRP11-eGFP cells using anti-GFP antibody. Co-immunoprecipitated proteins were identified by mass spectrometry. We obtained several putative NLRP11 interactors that were not detected in two independent control immunoprecipitation experiments conducted with the corresponding eGFP-expressing HeLa cell line (**Supplementary Table 1**). Due to its well-described functions in anti-viral innate immune signaling (51, 52, 68), we selected the DEAD-box protein DDX3X as the most interesting candidate for further analysis. To validate the interaction, we used a specific antibody directed against DDX3X. In independent experiments we could confirm the presence of endogenous DDX3X in co-immunoprecipitations from HEK293-NLRP11-eGFP cells, while it was absent in co-immunoprecipitations conducted with HEK293-eGFP control cells (**Figure 1D**). Unfortunately, due to lack of a specific antibody against human NLRP11, we could not assess the interaction with endogenous NLRP11.

To map the interaction domain for DDX3X in NLRP11, we expressed myc-tagged NLRP11 truncation mutants in HEK293T cells together with eGFP-DDX3X or empty vector and performed immunoprecipitations using anti-GFP antibody. We also confirmed binding of myc-NLRP11 to DDX3X in these experiments (**Figure 1E**). Deletion of the NLRP11 PYD did not influence binding, nor was the PYD sufficient to facilitate binding to DDX3X. While the LRR domain alone showed a strong interaction with DDX3X, we only observed limited binding of the NACHT domain to DDX3X (**Figure 1E**). This finding is in line with results obtained in our previous work, where we showed that the LRR domain of NLRP11 is sufficient for inhibition of TBK1-induced type I IFN (48).

**Figure 2:**
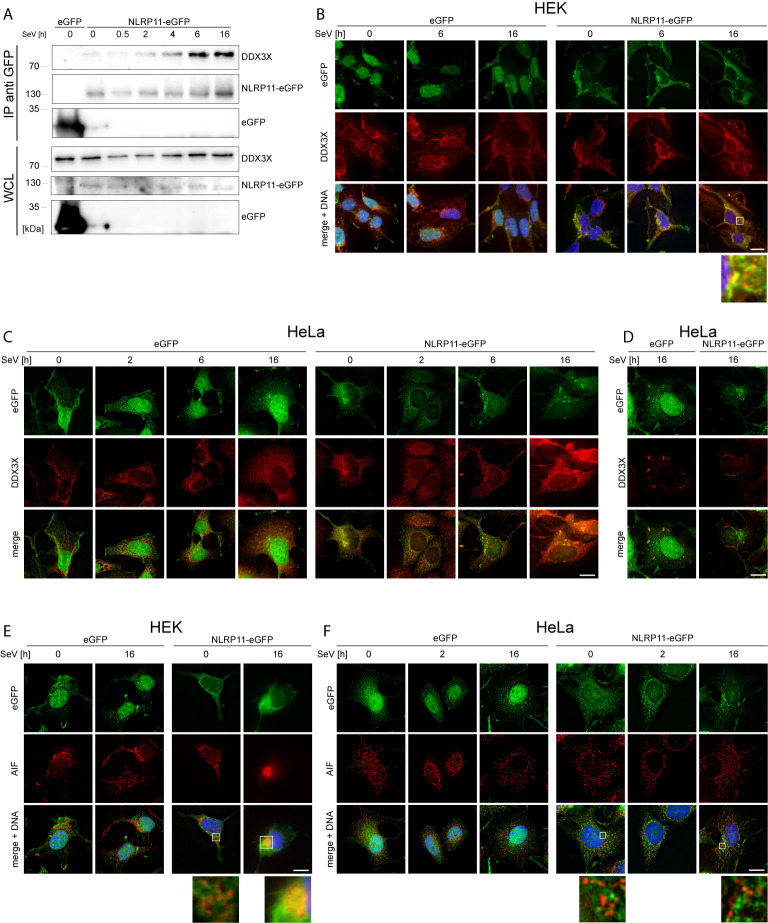
Interaction and co-localization of NLRP11 and DDX3X upon infection. **(A)** Co-immunoprecipitation of NLRP11-eGFP from HEK293-eGFP and HEK293-NLRP11-eGFP cells after induction with doxycycline overnight and infection with SeV for the indicated time. Immunoblots of the IPs and whole cell lysates (WCL) were probed for DDX3X and GFP. The blots are representative of two independent experiments. **(B–F)** Indirect immunofluorescence micrographs of HEK293 **(B, E)**, or HeLa- **(C, D, F)**, eGFP or NLRP11-eGFP cells induced with doxycycline overnight and infected with SeV for the indicated time. 3D deconvolution of DDX3X **(B–D)**, or AIF **(E, F)** staining (red), together with the eGFP signal (green) are shown. Nuclei are stained with Hoechst (blue). Scale bars = 10 µm.

We next set out to analyze the functional relevance of the DDX3X-NLRP11 interaction. First, we tested whether the NLRP11-DDX3X complex formation changed during Sendai virus (SeV) infection. Starting at 4 h post infection, we observed increased expression levels and co-immunoprecipitation of endogenous DDX3X with NLRP11-eGFP in HEK293 cells that was strongest at 6 h and 16 h post infection (**Figure 2A**). We next analyzed the subcellular localization dynamics of NLRP11 and DDX3X during infection with SeV by indirect immunofluorescence microscopy. In HEK293-NLRP11-eGFP cells we confirmed co-localization of NLRP11 and DDX3X in the cytosol. This co-localization was maintained and appeared slightly enhanced during SeV infection with more pronounced co-localization at 16 h post infection compared to steady state levels (**Figure 2B**). Co-localization of DDX3X and NLRP11-eGFP was also observed in HeLa-NLRP11-eGFP cells, where NLRP11-eGFP continued to stably co-localize with DDX3X over the course of 16 h of infection in the majority of cells (**Figure 2C**). However, NLRP11 was not recruited to distinct DDX3X clusters that appeared at 16 h post infection in a minority of cells (**Figure 2D**). The formation of these structures was also not influenced by the presence, or absence of NLRP11-eGFP (**Figure 2D**). These structures likely represent stress granules that DDX3X is known to be recruited into (60, 69, 70) and were only present in a minority of the cells. In line with data previously published by Qin et al. (49), HEK293-NLRP11-eGFP cells showed no co-localization of NLRP11-eGFP with the mitochondrial marker apoptosis-inducing factor (AIF) at steady state conditions, but we observed recruitment of NLRP11 to mitochondria at 16 h post infection (**Figure 2E**). In HeLa NLRP11-eGFP cells, NLRP11 was localized in proximity to mitochondria in untreated cells and this localization pattern persisted during 16 h of SeV infection, with partial co-localization observed at 16 h post infection (**Figure 2F**).

Taken together, we identified DDX3X as a novel binding partner of NLRP11. Mapping of the interaction domain in NLRP11 identified that the LRR region was sufficient for DDX3X binding. We further demonstrated that the interaction between NLRP11 and DDX3X occurred in the cytosol in proximity to mitochondria.

### NLRP11 Prevents the Post-Translational Modification of DDX3X by IKKϵ

**Figure 3:**
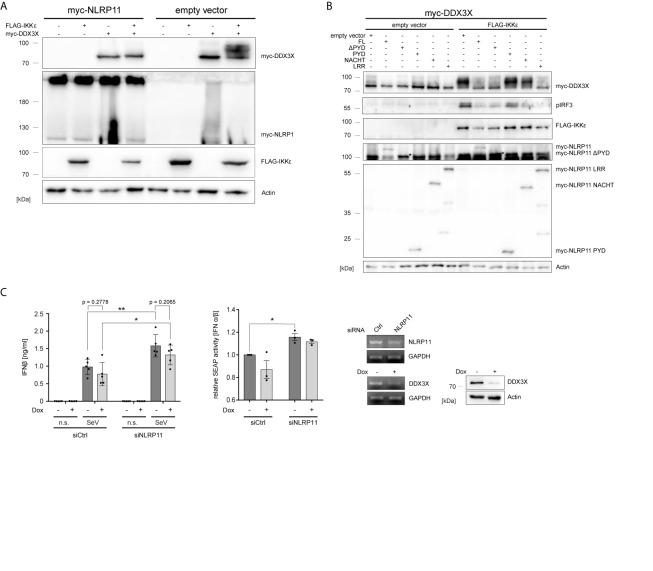
NLRP11 inhibits the phosphorylation of DDX3X by IKKϵ. **(A, B)** Immunoblot of whole cell lysates from HEK293T cells expressing FLAG-IKKϵ, myc-DDX3X and either myc-NLRP11 **(A)**, or myc-NLRP11 deletion constructs **(B)** as indicated. Blots were probed for myc, FLAG, pIRF3 and actin as loading control. Representative blots of at least two independent experiments are shown. *, myc-NLRP11 ΔPYD **(C)** IFNβ release from macrophage-like differentiated shDDX3 THP1 cells. DDX3X targeting shRNA was expressed by induction with doxycycline (Dox) for 48 h and NLRP11 was targeted by siRNA transfection for 72 h, as indicated. Cells were infected with SeV for 16 h. IFNβ quantification by ELISA (left graph) and bioassay (right graph) are shown as means ± SEM (IFNβ: n = 5, SEAP: n = 3, ± SEM). *p < 0.05; **p < 0.01 Welch’s, two-sided t-test. Knock-down efficiency of NLRP11 and DDX3X was validated by endpoint PCR, and protein levels of DDX3X by immunoblot (upper panels). Immunoblots were probed for DDX3X and actin as loading control. n.s., not stimulated.

Gu et al. reported that IKKϵ can phosphorylate DDX3X and that this is a prerequisite for the interaction of DDX3X with IRF3 and subsequent activation of the *Ifnb* promotor (54). We therefore investigated whether NLRP11 influences this posttranslational modification of DDX3X. As shown before, co-expression of IKKϵ and DDX3X in HEK293T cells induced a change in the electrophoretic mobility of DDX3X, which is indicative of phosphorylation (54). This up-shift of DDX3X was clearly suppressed by co-expression of NLRP11 (**Figure 3A**).

We have previously shown that the ability of NLRP11 to inhibit TBK1-induced IFNβ production is dependent on its LRRs (48). Given that the LRR region also interacted with DDX3 (**Figure 1E**), we tested whether this domain is required and sufficient to inhibit IKKϵ-mediated DDX3X phosphorylation and, consequently, downstream activation of IRF3 (54). Expression of full-length NLRP11, NLRP11-ΔPYD and NLRP11-LRR reduced the IKKϵ-induced upshift of DDX3X, while this was not observed for the PYD or NACHT domain of NLRP11. Consistent with this data, activation of IRF3, assessed by serine 396 phosphorylation, was also strongly reduced when NLRP11 full length, ΔPYD or the LRRs were expressed (**Figure 3B**).

Next, in order to interrogate the consequences of the DDX3X-NLRP11 interaction on IFNβ induction, we performed siRNA-mediated knock-down of NLRP11 in macrophage-like differentiated human THP1 cells in which DDX3X expression can be suppressed by Tet-inducible expression of a specific short hairpin RNA (shRNA) (THP1 shDDX3X) (64). THP1 cells were used for these experiments because they express higher levels of endogenous NLRP11 and IKKϵ compared to HeLa and HEK293T cells (48). In accordance with recent data (48, 49), knock-down of NLRP11 led to significantly increased IFNβ production in response to SeV infection (**Figure 3C**). As reported previously (51), shRNA-mediated knock-down of DDX3X had the opposite effect and reduced SeV-induced IFNβ expression (**Figure 3C**). However, DDX3X depletion resulted in a similar ratio of IFNβ reduction compared to control (-Dox) in both siCtrl cells and siNLRP11 treated cells (**Figure 3C**). Qualitatively similar results were obtained when measuring IFNβ activity in a bioassay (**Figure 3C**).

Overall, our data suggest that NLRP11 represses type I interferon responses by affecting IKKϵ-mediated posttranslational modification of DDX3X.

### NLRP11 Counteracts the Effect of DDX3X on NLRP3 Inflammasome Activation

**Figure 4:**
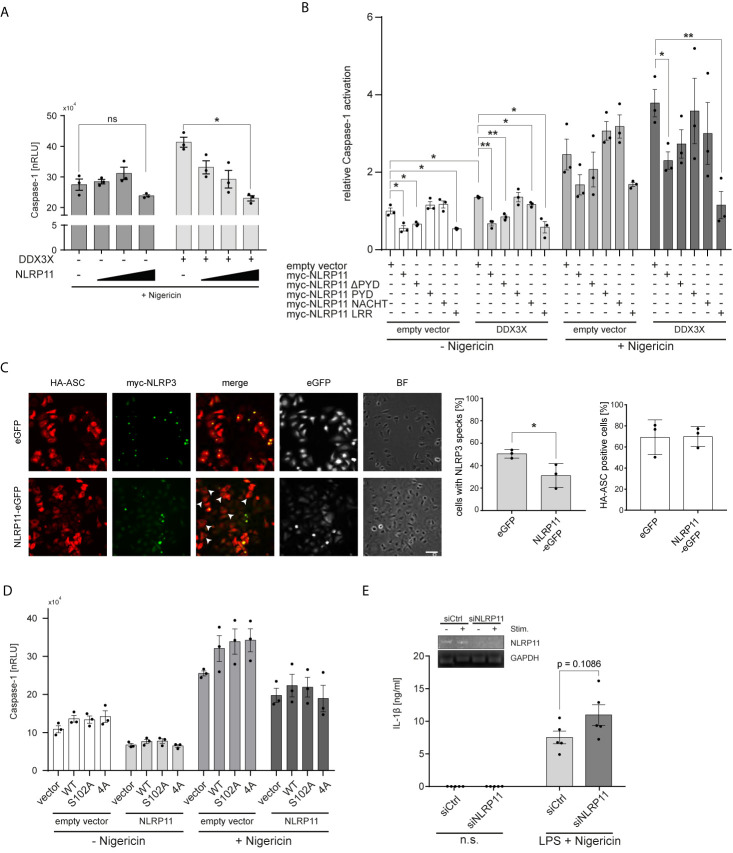
NLRP11 suppresses NLRP3 inflammasome activation by DDX3X. **(A, B)** iGLuc caspase-1 reporter assay in HEK293T cells expressing myc-NLRP3 (15 ng), HA-ASC, and caspase-1 (10 ng each) together with the indicated proteins. Cells were treated with 15 µM nigericin for 3 h. Means of three independent experiments (each conducted in triplicates) ± SEM are shown. *p < 0.05; **p < 0.01 Welch’s, two-sided t-test. **(B)**, values relative to the mean of three independent experiments conducted in triplicates of cells, transfected with the reporter plasmid, NLRP3, ASC and caspase-1, not treated with nigericin, are shown. **(C)** Indirect immunofluorescent micrographs of HeLa-eGFP or HeLa-NLRP11-eGFP cells, induced with doxycycline overnight and transfected with HA-ASC and myc-NLRP3. Staining for HA and myc, as well as eGFP-signal is shown. Images representative of three independent experiments are shown. Scale bar = 50 µm. HA-ASC expressing cells without speck-formation are indicated with white arrow heads. Right panels: Quantification of cells with myc-NLRP3 specks and quantification of cells with HA-ASC staining (blinded counting of 150 cells per condition from n = 3). **(D)** iGLuc caspase-1 reporter assay in HEK293T cells expressing myc-NLRP3 (15 ng), HA-ASC, and caspase-1 (10 ng each) together with the indicated proteins. Cells were treated with 15 µM nigericin for 3 h. Means of three independent experiments (each conducted in triplicates) ± SEM are shown. **(E)** IL-1β release from macrophage-like differentiated THP1 cells after 72 h of siRNA-mediated knock-down. Cells were primed for 4 h with 100 ng/ml LPS followed by stimulation with 10 µM nigericin for 2 h. Means of five independent experiments ± SEM are shown. *Inlay:* endpoint PCR for NLRP11 and GAPDH of a representative experiment for validation of knock-down efficiency. n.s., not stimulated.

Considering the recent identification of DDX3X as a positive regulator of NLRP3 inflammasome formation (60), we next investigated whether NLRP11 affects DDX3X’s function in this context. We previously showed that NLRP11 cannot induce caspase-1 activation itself, nor does NLRP11 recruit the inflammasome adaptor ASC (48), suggesting that NLRP11 does not form a classical inflammasome when ectopically expressed in cells. Instead, we observed a trend towards reduced caspase-1 activation when NLRP11 was overexpressed (48). We therefore now investigated whether NLRP11 interferes with NLRP3 inflammasome activation, conceivably *via* sequestration of DDX3X. In line with the report from the Kanneganti lab (60), DDX3X enhanced nigericin-induced pro-caspase-1 cleavage, as measured by the iGLuc reporter assay, where a luciferase protein gets activated by caspase-1 cleavage in HEK293T cells (61) (**Figure 4A**). Expression of increasing amounts of myc-NLRP11 did not significantly affect NLRP3 inflammasome activation in the absence of exogenous DDX3X expression but led to a dose-dependent reduction of caspase-1 activation back to baseline levels in DDX3X overexpressing cells (**Figure 4A**).

To determine whether the LRRs of NLRP11 were involved in the negative regulation of the NLRP3 inflammasome, we performed caspase-1 activation assays with our different NLRP11 truncation mutants. Expression of full-length NLRP11 or the LRRs led to a significant reduction of nigericin-induced caspase-1 activation. The same trend was observed in cells expressing only endogenous DDX3X (**Figures 4A, B**). NLRP11, NLRP11-ΔPYD and NLRP11-LRRs also caused a significant decrease in baseline caspase-1 activity (in the absence of nigericin) both in the presence and absence of exogenous DDX3X (**Figure 4B**). Taken together, this data shows that the LRRs of NLRP11 are both necessary and sufficient to dampen NLRP3 inflammasome activation, which might be a result of DDX3X recruitment and sequestration *via* the LRR domain of NLRP11. To provide further evidence we performed ASC speck-formation assays. When we co-expressed HA-ASC together with myc-NLRP3 in our HeLa cell lines, we found fewer ASC specks in NLRP11-eGFP-expressing cells compared to eGFP-expressing cells that served as control. Quantitative analysis revealed a reduction from about 51% speck-containing cells for HeLa-eGFP cells to about 31% for HeLa-NLRP11-eGFP cells (**Figure 4C**). Equal transfection efficiency of both cell lines was confirmed by blinded counting of HA-ASC positive cells (**Figure 4C**). This data strongly suggests that NLRP11 can inhibit assembly of NLRP3 inflammasomes.

In **Figures 3A, B**, we showed that NLRP11 suppresses IKKϵ-mediated phosphorylation of DDX3X. We next asked whether this phosphorylation plays a role in the regulation of NLRP3 inflammasome activation by DDX3X. To this end, we performed iGLuc reporter assays with the S102A mutant of DDX3X lacking the IKKϵ-phosphorylation site shown to be critical for IRF3 recruitment to DDX3X and IFNβ induction. We also tested another DDX3X mutant in which three further IKKϵ-phosphorylation sites in the N-terminus of DDX3X (S71A, S82A, S83A) are mutated in addition to S102 (54). We did not observe any differences in the capacity of these DDX3X mutants to enhance caspase-1 activation both in presence and absence of NLRP11 expression (**Figure 4D**), suggesting that these DDX3 phosphorylation events do not regulate its effect on inflammasome formation.

Finally, to corroborate a negative regulatory role for NLRP11 in inflammasome induced caspase-1 activation, we knocked down endogenous NLRP11 expression in macrophage-like differentiated THP1 cells using a specific siRNA (48). We first primed the differentiated THP1 cells with LPS and then induced NLRP3 inflammasome activation with nigericin. IL-1β secretion, a well-established read-out for NLRP3 inflammasome activation, was measured. Knock-down of NLRP11 resulted in increased IL-1β secretion (**Figure 4E**), albeit this effect was not significant (p=0.1086).

Taken together, these data provide evidence that NLRP11 can negatively regulate NLRP3 inflammasome activation and suggest that this is mediated via its interaction with DDX3X.

## Discussion

**Figure 5:**
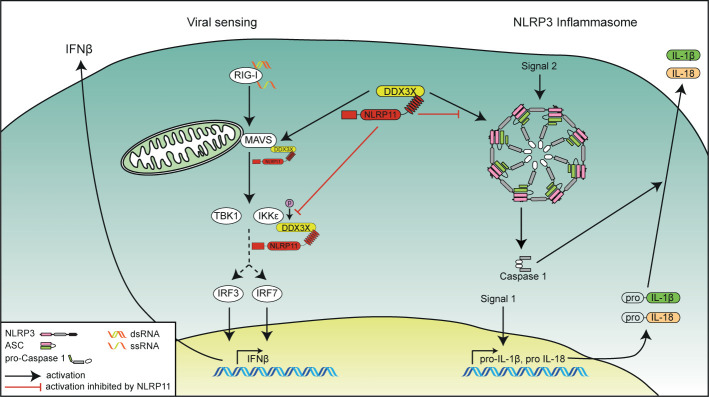
Schematic overview of the functional interplay of DDX3X and NLRP11 in innate immunity. The role of NLRP11 in attenuating RIG-I-induced type I interferon responses (left side) and in the activation of the NLRP3 inflammasome (right side) is depicted. NLRP11 interacts with DDX3X *via* its LRR domain. NLRP11 reduces type I interferon responses downstream of RIG-I activation by negative regulation of the activity of DDX3X in type I interferon induction. NLRP11 can also interfere with DDX3X-mediated NLRP3 inflammasome activation, leading to reduced IL-1β processing.

Tight control and coordinated resolution of pro-inflammatory signaling pathways is an essential part of the immune response. While insufficient activation of innate immunity might provide pathogens the opportunity to thrive, an overshooting immune response can result in immunopathology. Many control mechanisms have therefore evolved that meticulously regulate the activation level of innate immune responses. We and others previously showed that NLRP11 can act as a negative regulator of antiviral type I IFN expression (48, 49) and NF-κB-dependent pro-inflammatory cytokine responses (47). Here we expand the mechanistical understanding of NLRP11’s regulation of antiviral responses by showing that it interacts with and inhibits the DEAD-box protein DDX3X (**Figure 5**). DDX3X has previously been shown to enhance the RIG-I-mediated antiviral response at multiple levels (50–54). Interestingly, other DEAD-box helicases have also been shown to form complexes with NLR family members: In mice, Nlrp9b uses the DEAD-box protein Dhx9 as a sensor for double-stranded RNA to induce inflammasome activation and pyroptosis following infection with dsRNA viruses (71). Dhx15 has also been shown to sense viral RNA and to bind to Nlrp6, mediating its interaction with MAVS (72). Sensing of viral and bacterial RNA by DHX33 has been shown to induce an interaction with NLRP3, enhancing inflammasome formation and caspase-1 activation (73). The latter interaction was shown to be dependent on the NACHT domain of NLRP3. Similarly, the DDX3X interaction with NLRP3 was also mediated by the NACHT domain, resulting in enhanced activation of caspase-1 (60). In contrast, we demonstrated that the interaction between NLRP11 and DDX3X is mediated by the LRRs of NLRP11. This difference in binding domains might explain why some interactions between DExD/H-box proteins and NLR proteins result in increased activation of immune signaling pathways, whereas the interaction we describe here has a negative regulatory effect. The LRR domain of NLRP11 has already been shown to inhibit type I IFN induction upon viral challenge (48, 49). Here we show that the LRR domain is also sufficient to inhibit hyperphosphorylation of DDX3X induced by IKKϵ (51, 54). This supports our hypothesis that NLRP11’s repression of IFN induction is at least partially mediated by targeting DDX3X.

DDX3X is known to positively regulate the RIG-I pathway by interacting with multiple downstream signaling molecules. RIG-I signals *via* its mitochondrial adaptor protein MAVS to induce type I IFN transcription (50). Previously, NLRP11 was shown to be recruited to MAVS *via* its LRRs to modulate TRAF6 function and stability (49). However, no direct physical interaction between NLRP11 and MAVS was shown in this study. This raises the possibility that DDX3X could be involved in mediating this NLRP11-MAVS interaction. In our HEK293-NLRP11-eGFP cell line, we confirmed recruitment of NLRP11 to mitochondria 16 h post infection, as visualized by co-staining with AIF, however, the cellular morphology was heavily disturbed after 16 h of virus infection. The physiological relevance of the change in subcellular localization of NLRP11 in this cell type thus remains elusive.

Surprisingly, NLRP11 knock-down still increased IFNβ induction in DDX3X knock-down cells (**Figures 3C, D**). However, our DDX3X knock-down was only partial as DDX3X is required for cell viability, and therefore residual DDX3X protein levels might have affected the outcome of this experiment. It is also possible, that the NLRP11-DDX3X interaction more strongly perturbs the function of IKKϵ in ISG induction (74, 75) than IFNβ induction which is more TBK1-mediated (23, 76). This would explain why NLRP11 interferes with antiviral signaling at multiple steps downstream of RIG-I, enabling it to negatively regulate expression of both IFN (47–49) and ISGs that are directly regulated by IKKϵ.

In addition to this role in regulating antiviral gene expression, we provide evidence that NLRP11 can act as a negative regulator of the NLRP3 inflammasome. We confirmed that DDX3X is a positive regulator of the NLRP3 inflammasome, as reported recently (60) and show that expression of NLRP11 counteracts this effect of DDX3X. We also observed a trend towards higher IL-1β secretion upon NLRP11 knock-down in macrophage-like differentiated THP1 cells, but the rather low NLRP11 expression levels in THP1 cells (48) might limit the effect of siRNA-mediated knock-down.

The PYD domain of NLRP11 was not required for blocking the DDX3X effect on the NLRP3 inflammasome, instead expression of the NLRP11 LRRs was sufficient. Samir et al. proposed that recruitment of DDX3X by NLRP3 is critical for functional inflammasome formation (60). This is in line with our data, which suggests that NLRP11 binding to DDX3X *via* its LRRs reduces caspase-1 activation. It would be interesting whether this effect results from DDX3X sequestration by NLRP11 or competition between NLRP3 and NLRP11 for binding sites on DDX3X. When overexpressing NLRP11-eGFP in HeLa cells, we did not observe an obvious change in the subcellular localization of endogenous DDX3X, arguing against the sequestration mechanism. Another possibility is that NLRP11 inhibits posttranslational modifications of DDX3X that are important for its involvement in the NLRP3 inflammasome. This hypothesis is based on our finding that the NLRP11 LRRs inhibited the NLRP3 inflammasome and prevented IKKϵ-mediated hyperphosphorylation of DDX3X. Although we were unable to implicate the phosphorylation sites of DDX3X that are involved in IRF3 activation (54) in its regulatory effect on the NLRP3 inflammasome, it is likely that other DDX3X post-translational modifications could also be affected by NLRP11.

In summary, we show that NLRP11 is an NLR family member that negatively regulates NLRP3 inflammasome activity and interferes with the induction of antiviral type I IFN. For both regulatory effects, we identified a novel role for DDX3X, which we discovered as a novel binding partner of NLRP11, putting a further spotlight on this interesting DEAD-box protein.

## Data Availability Statement

The original contributions presented in the study are included in the article/**Supplementary Material**. Further inquiries can be directed to the corresponding author.

## Author Contributions

IK, SB, and CG performed the experiments and analyzed data. JP performed MS analysis and data interpretation. MS provided reagents. NM provided essential conceptional input and edited the manuscript. IK, MS, and TK wrote and edited the manuscript and figures. MS and TK conceptualized the study, analyzed data, and assured funding. All authors contributed to the article and approved the submitted version.

## Funding

IK acknowledges support by the Landesgraduiertenförderung of Baden-Württemberg. Research in the MS laboratory was funded by Science Foundation Ireland and the Irish Health Research Board.

## Conflict of Interest

The authors declare that the research was conducted in the absence of any commercial or financial relationships that could be construed as a potential conflict of interest.
