# Supplementary material for: DDX3X Links NLRP11 to the Regulation of Type I Interferon Responses and NLRP3 Inflammasome Activation
Source: Front Immunol. 2021 May 13;12:653883. doi: 10.3389/fimmu.2021.653883 (PMC8158815; doi:10.3389/fimmu.2021.653883)
Supplement: Supplementary file 1 [file DataSheet_1.pdf]

Supplementary Material

Supplementary Table 1: List of proteins that co-purified with NLRP11-eGFP. Proteins identified by nanoLC-ESI-MS/MS in two independent co-immunoprecipitation experiments (Exp1, Exp2) without any peptide hits in the control immunoprecipitation (eGFP) are shown.

**Protein Name**	**Gene Name**	**Unique Peptides**	
		Exp1	Exp2
NACHT, LRR and PYD domains-containing protein 11	NLRP11	38	50
ATP-binding cassette subfamily D member 3	ABCD3	1	1
Alpha-actinin 1	ACTN1	2	1
Fatty aldehyde dehydrogenase	ALDH3A2	2	2
Acidic leucine-rich nuclear phosphoprotein 32 family member A	ANP32A	1	2
Large proline-rich protein BAG 6	BAG6	1	8
Brain acid soluble protein 1	BASP1	5	2
ATP-dependent RNA helicase DDX3X	DDX3X	1	2
DnaJ homolog subfamily B member 1	DNAJB1	1	7
Guanine nucleotide-binding protein G(s) subunit alpha isoforms XLas	GNAS	10	4
Guanine nucleotide-binding protein G(I)/G(S)/G(T) subunit beta-2	GNB2	11	3
Guanine nucleotide-binding protein G(I)/G(S)/G(O) subunit gamma-12	GNG12	4	1
HCLS1-associated protein X-1	HAX-1	2	3
Heat shock protein 105 kDa	HSPH1	1	8
26S proteasome regulatory subunit 8	PSMC5	1	4
26S proteasome non-ATPase regulatory subunit 1	PSMD1	3	5
26S proteasome non-ATPase regulatory subunit 2	PSMD2	1	6
26S proteasome non-ATPase regulatory subunit 3	PSMD3	1	3
26S proteasome non-ATPase regulatory subunit 6	PSMD6	1	1
26S proteasome non-ATPase regulatory subunit 11	PSMD11	2	5

26S proteasome non-ATPase regulatory subunit 13	PSMD13	2	2
26S proteasome non-ATPase regulatory subunit 14	PSMD14	1	6
Dolichyl-diphosphooligosaccharide-protein glycosyltransferase subunit 2	RPN2	1	4
40S ribosomal protein S24	RPS24	2	2
Reticulon-4	RTN4	1	1
Sequestosome 1	SQSTM1	3	5
Stress-induced-phosphoprotein 1	STIP1	14	27
E3 ubiquitin protein ligase CHIP	STUB1	8	10
Transketolase	TKT	1	1
Tubulin beta-2A chain	TUBB2A	1	1
Ubiquitin-like protein 4A	UBL4A	2	7
